# Evidence of capsaicin synthase activity of the *Pun1*-encoded protein and its role as a determinant of capsaicinoid accumulation in pepper

**DOI:** 10.1186/s12870-015-0476-7

**Published:** 2015-03-28

**Authors:** Kana Ogawa, Katsunori Murota, Hanako Shimura, Misaki Furuya, Yasuko Togawa, Takeshi Matsumura, Chikara Masuta

**Affiliations:** Graduate School of Agriculture, Hokkaido University, Sapporo, 060-8589 Japan; Plant Molecular Technology Research Group, Research Institute of Bioproduction, National Institute of Advanced Industrial Science and Technology, Sapporo, 062-8517 Japan

**Keywords:** *Capsicum*, pAMT, Pun1, Protoplast assay, Pungency, Vanillylamine, Virus-induced gene silencing

## Abstract

**Background:**

Capsaicinoids, including capsaicin and its analogs, are responsible for the pungency of pepper (*Capsicum* species) fruits. Even though capsaicin is familiar and used daily by humans, the genes involved in the capsaicin biosynthesis pathway have not been well characterized. The putative aminotransferase (pAMT) and Pungent gene 1 (Pun1) proteins are believed to catalyze the second to last and the last steps in the pathway, respectively, making the Pun1 protein the putative capsaicin synthase. However, there is no direct evidence that Pun1 has capsaicin synthase activity.

**Results:**

To verify that the Pun1 protein actually plays a role in capsaicin production, we generated anti-Pun1 antibodies against an *Escherichia coli*-synthesized Pun1 protein and used them to antagonize endogenous Pun1 activity. To confirm the anti-Pun1 antibodies’ specificity, we targeted *Pun1* mRNA using virus-induced gene silencing. In the *Pun1*-down-regulated placental tissues, the accumulated levels of the Pun1 protein, which was identified on a western blot using the anti-Pun1 antibodies, were reduced, and simultaneously, capsaicin accumulations were reduced in the same tissues. In the *de novo* capsaicin synthesis *in vitro* cell-free assay, which uses protoplasts isolated from placental tissues, capsaicin synthesis was inhibited by the addition of anti-Pun1 antibodies. We next analyzed the expression profiles of *pAMT* and *Pun1* in various pepper cultivars and found that high levels of capsaicin accumulation always accompanied high expression levels of both *pAMT* and *Pun1*, indicating that both genes are important for capsaicin synthesis. However, comparisons of the accumulated levels of vanillylamine (a precursor of capsaicin) and capsaicin between pungent and nonpungent cultivars revealed that vanillylamine levels in the pungent cultivars were very low, probably owing to its rapid conversion to capsaicin by Pun1 soon after synthesis, and that in nonpungent cultivars, vanillylamine accumulated to quite high levels owing to the lack of Pun1.

**Conclusions:**

Using a newly developed protoplast-based assay for *de novo* capsaicin synthesis and the anti-Pun1 antibodies, we successfully demonstrated that the *Pun1* gene and its gene product are involved in capsaicin synthesis. The analysis of the vanillylamine accumulation relative to that of capsaicin indicated that Pun1 was the primary determinant of their accumulation levels.

**Electronic supplementary material:**

The online version of this article (doi:10.1186/s12870-015-0476-7) contains supplementary material, which is available to authorized users.

## Background

Capsaicin is the compound responsible for the pungency of peppers [1]. Capsaicin and its analogs are called capsaicinoids, and the fundamental structure of most capsaicinoids is a branched-chain fatty acid amide of vanillylamine. Capsaicinoids, produced only by species of the genus *Capsicum*, are specifically synthesized in placental tissues, mostly between 20 and 30 days after flowering (daf) in the pepper fruits of pungent cultivars [2]. Capsaicinoids are used in the human diet for their distinct pungent taste and bioactivities, such as thermogenesis [3,4] and the suppression of fat accumulation [5]. In addition, they are used for pharmaceutical purposes because they have potential bioactivities that are ascribed to antioxidants [6] and anti-cancer agents [7]. Although pungency in peppers is a desirable trait, nonpungent cultivars also have been developed as vegetables. Nonpungent cultivars have been shown either to accumulate very few capsaicinoids or to accumulate capsinoids, such as capsiate, which are capsaicinoid analogs (branched-chain fatty acid ester of vanillyl alcohol) that lack the stimuli of the capsaicinoids [8].

Two pathways are involved in capsaicin biosynthesis: (1) the phenylpropanoid pathway derived from phenylalanine leading to vanillylamine; and (2) the branched-chain fatty acid pathway derived from valine leading to 8-methyl-6-nonenoyl-CoA [9-11]. The condensation reaction of vanillylamine with 8-methyl-6-nonenoyl-CoA is thought to be catalyzed by a coenzyme A-dependent acyltransferase. Although the genes involved in capsaicinoid synthesis have been extensively studied, the gene responsible for the acylation of vanillylamine remained unknown until Kim *et al.* [12] reported the SB2-66 clone as a possible candidate. Stewart *et al.* [13] finally showed that SB2-66 was the putative acyltransferase involved in capsaicinoid production and encoded by *AT3* gene, namely *Pungent gene 1* (*Pun1*) as capsaicin synthase (CS). The expression profile of *Pun1* correlates with pepper pungency and the deletion or down-regulation of the *Pun1* gene results in a decreased accumulation of capsaicinoids. In addition to capsaicinoids, *Pun1* has been reported to control capsinoid synthesis in nonpungent pepper cultivars [14,15].

Another important step in capsaicin biosynthesis is the conversion of vanillin to vanillylamine, and a putative aminotransferase (pAMT) has been proposed to be the enzyme responsible for vanillin’s transamination. The full-length cDNA clone of *pAMT* was identified from a cDNA library by differential display [16]. Abraham-Juárez *et al.* [17] showed reduced capsaicinoid levels using virus-induced gene silencing (VIGS) against *pAMT*, providing evidence that the putative *pAMT* gene was involved in capsaicinoid biosynthesis. In addition, Lang *et al.* [18] reported that the capsaicinoids were poorly synthesized in a spontaneous mutant, cultivar ‘CH-19 Sweet’, and that this phenotype was due to the loss-of-function of the *pAMT* gene in the particular mutant. Using this mutant, Tanaka *et al.* [19] analyzed the defective *pAMT* gene in detail and found that a single amino acid substitution was responsible for the capsaicin deficiency.

Very recently, pAMT was demonstrated, using an *Escherichia coli*-expressed recombinant enzyme, to act as a vanillin aminotransferase [20]. However, *Pun1* is considered a putative gene because its encoded enzyme has not been biochemically analyzed, even though functional genomics studies indicate that *Pun1* is responsible for the acylation of vanillylamine and vanillyl alcohol in *Capsicum sp*. [13-15]. In the present study, we used protoplasts and anti-Pun1 antibodies to verify that the *Pun1* gene is actually involved in the crucial step of capsaicin biosynthesis. In addition, we investigated the expression profiles for two important genes, *pAMT* and *Pun1*, in capsaicin biosynthesis and discussed the roles of pAMT and Pun1 in various pepper cultivars that differ in pungency.

## Results

### Preparation of anti-Pun1 antibodies against the Pun1 protein to antagonize endogenous Pun1 activity in an *in vitro* assay

**Figure 1:**
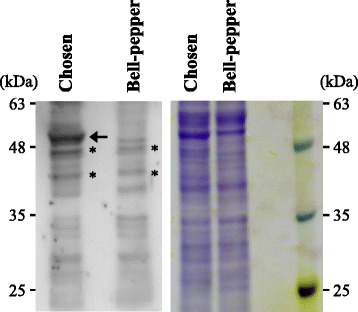
**Western blot analysis of the Pun1 protein in the pungent variety ‘Chosen’ and the nonpungent bell pepper using anti-Pun1 antibodies.** As indicated by the arrow, the Pun1 protein was detected in the total proteins isolated from placental tissues of ‘Chosen’ by polyclonal antibodies against the *E. coli*-synthesized Pun1 protein (left). The Coomassie brilliant blue-stained gel is shown as a loading control (right). Asterisks show nonspecific bands.

**Figure 2:**
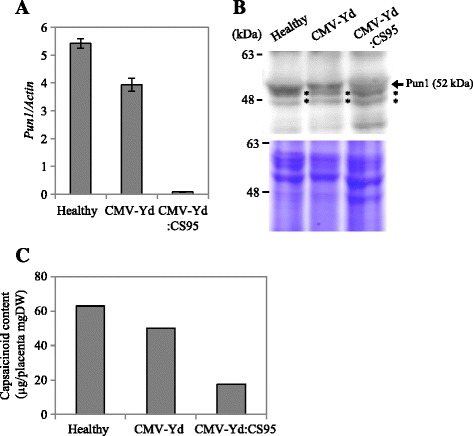
**Effect of virus-induced gene silencing against the** ***Pun1*** **gene in pepper placental tissues. (A)** *Pun1* mRNA accumulation levels in placental tissues infected with CMV-Yd:CS95. CMV-Yd and CMV-Yd:CS95 are the empty vector and the vector containing a 95-nt sequence of the *Pun1* gene, respectively. The *Pun1* mRNA levels are shown relative to the *Actin* mRNA level in placental tissues that were all harvested at 20 to 25 days after flowering. Values are means ± SD obtained from three replicates. **(B)** Western blot analysis of the Pun1 protein in CMV-Yd:CS95-infected placental tissues. The Coomassie brilliant blue -stained gel containing the same proteins as in the blot is shown as a loading control. Asterisks indicate nonspecific bands. **(C)** Capsaicinoid accumulation levels in CMV-Yd:CS95-infected placental tissues evaluated by HPLC. The same tissues were used for HPLC analysis as those used for western blotting.

To verify that the Pun1 protein (syn. AT3) is the actual acyltransferase for capsaicin production, we first developed an *in vitro* assay system for capsaicin synthesis using recombinant Pun1 proteins, which are produced from the cDNA clone of *Pun1* inserted into an *E. coli* expression vector. However, in preliminary experiments, we were not able to use recombinant Pun1 proteins in the enzymatic activity assay because they were mostly insoluble when expressed in *E. coli*, as reported previously [13]. Alternatively, we produced anti-Pun1 antibodies using the *E. coli*-synthesized Pun1 protein and tried to use them as antagonists of endogenous Pun1 activity in the *in vitro* assay system. To test the specificity of the created antibodies for the Pun1 protein, we first conducted a western blot analysis using total proteins from a nonpungent bell pepper, which is defective in the *Pun1* gene, as a true negative control. As shown in Figure 1, we detected a very strong band in proteins from a pungent cultivar (‘Chosen’) at the expected size of 52 kDa, whereas there was no 52 kDa-signal in the bell pepper. We tested whether the antibodies cross-react with another acyltransferase, hydroxycinnamoyl transferase (HCT), which is also listed as a candidate enzyme in the capsaicinoid synthesis pathway [21,22]. The cDNA of the pepper HCT (EU616565) with the FLAG tag sequence at the 3′ end was polymerase chain reaction (PCR)-amplified and then synthesized as the HCT-FLAG protein from *in vitro*-transcribed HCT-FLAG mRNA in a wheat germ system. The HCT-FLAG protein was clearly detected by an anti-FLAG antibody, but not by the anti-Pun1 antibodies, suggesting that the anti-Pun1 antibodies did not cross-react with the pepper HCT (Additional file 1: Figure S1). To further confirm the antibody specificity, we used VIGS to specifically reduce the expression level of *Pun1* mRNA and analyzed the capsaicinoid accumulation in the virus-infected pepper placenta. A 95-nt sequence of *Pun1* was inserted in the *Cucumber mosaic virus* (CMV) vector (CMV-Yd:CS95) to induce RNA silencing against *Pun1* mRNA. The pepper fruits infected with the virus vector containing the *Pun1* insert showed no apparent differences from those infected with the empty vector. Compared with healthy fruits, the virus-infected fruits were a little smaller but no great differences were observed. The quantitative reverse-transcription (RT)-PCR analysis confirmed that the *Pun1* mRNA levels were greatly reduced in the CMV-Yd:CS95-infected placental tissues (Figure 2A) in which the capsaicin accumulation was significantly reduced (Additional file 2: Figure S2). We next examined the Pun1 protein accumulation levels using a western blot analysis with the anti-Pun1 antibodies. As shown in Figure 2B, the Pun1 accumulation levels were indeed reduced in the CMV-Yd:CS95-infected placental tissues compared with those of the healthy and CMV-Yd-infected control tissues. These results suggest that the *Pun1* expression was specifically reduced by VIGS, and that the anti-Pun1 antibodies could detect the reduced levels of the Pun1 protein in a western blot. Consistent with those results, the capsaicinoid content in the same placental tissues infected with CMV-Yd:CS95 was also reduced to between ~25% and 33% of the control content (Figure 2C). The reduced level of the Pun1 protein detected by our antibodies reasonably reflected the reduced levels of capsaicinoid, and thus, we concluded that the specificity of the antibodies for the Pun1 protein was satisfactory.

### Evidence that the *Pun1*-encoded enzyme can catalyze capsaicin synthesis

**Figure 3:**
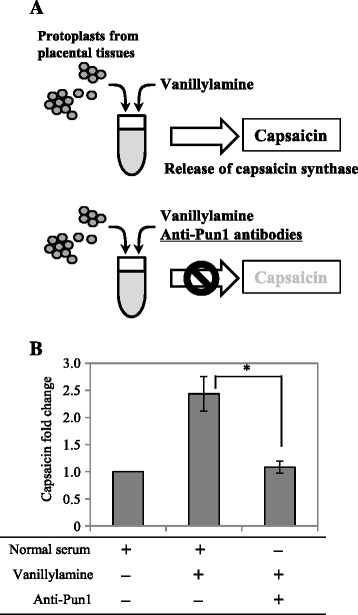
**Quantification of** ***de novo*** **-synthesized capsaicin in protoplasts treated with or without anti-Pun1 antibodies. (A)** Strategy for the cell-free assay of capsaicin synthesis. Protoplasts prepared from placental tissues of pungent peppers were opened in a hypotonic solution, including vanillylamine as a substrate, thus releasing enzymes immediately converted the exogenously added substrate to capsaicin using the endogenous 8-methyl-6-nonenoyl-CoA. Anti-Pun1 antibodies would inhibit this reaction by binding to the Pun1 protein. **(B)** Inhibition of capsaicin synthesis by anti-Pun1 antibodies. In the cell-free assay using protoplasts, *de novo*-synthesized capsaicin was analyzed in HPLC and quantified using a capsaicin standard calibration curve. The graph shows the data as fold change compared with the control [means ± SD relative to a control reaction treated with normal serum (set to 1.0) derived from four separate experiments]. The reduction in capsaicin synthesis in protoplasts with anti-Pun1 antibodies was statistically significant compared with control protoplasts using Student’s t-test. Asterisks indicate a significant difference at 0.05. A representative HPLC chromatogram is shown in Additional file 3: Figure S3.

As described above, we produced anti-Pun1 antibodies using the *E. coli*-synthesized Pun1 protein and used the antibodies as antagonists of Pun1 activity. To develop an *in vitro* assay system for capsaicin synthesis, we first prepared cell-free crude enzyme extracts from the placental tissue of a pungent pepper and then added vanillylamine. However, we were not able to detect any *de novo*-synthesized capsaicinoid, probably owing to an unknown inhibitor(s). We therefore isolated protoplasts from placental tissues and used them for the assay (Figure 3A). In this assay, CS would be released from the protoplasts soon after the cells are broken, which occurs when vanillylamine is added. In the protoplast-based assay, we were able to detect *de novo*-synthesized capsaicin (Additional file 3: Figure S3). Capsaicinoid synthesis requires 8-methyl-6-nonenoyl-CoA, as well as vanillylamine, as substrates, but 8-methyl-6-nonenoyl-CoA is not commercially available. In our protoplast-based assay, 8-methyl-6-nonenoyl-CoA was provided from the placental protoplasts, leading to capsaicin synthesis. Using this assay system, we analyzed whether the addition of the anti-Pun1 antibodies to the reaction would prevent *de novo* capsaicinoid synthesis. As we expected, the addition of anti-Pun1 antibodies significantly reduced capsaicin synthesis to less than half of the control without anti-Pun1 antibodies (Figure 3B, Additional file 3: Figure S3), suggesting that the Pun1 protein plays an essential role in capsaicin synthesis.

### Comparisons of expression levels of *pAMT* and *Pun1* between pungent and nonpungent cultivars

**Figure 4:**
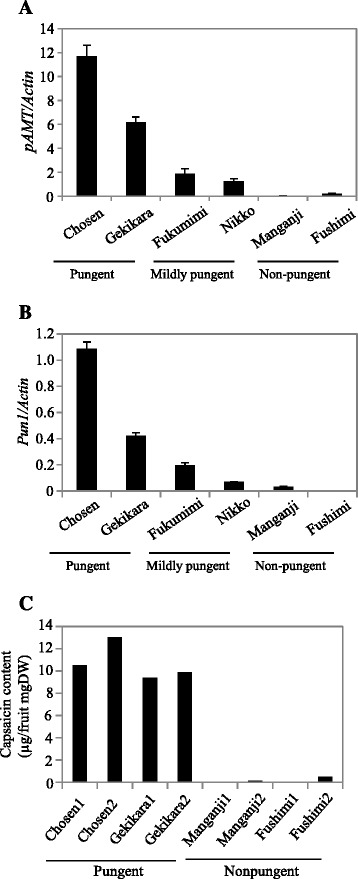
**Relationships between the expression levels of** ***pAMT*** **and** ***Pun1*** **genes, and the accumulation of capsaicin in different pepper cultivars. (A)** *pAMT* expression in different pepper cultivars. RNA transcript levels for the *pAMT* gene at 25 days after flowering in placental tissues were determined using quantitative RT-PCR (mean ± SE; *n* = 3). ‘Chosen’ and ‘Gekikara’: pungent pepper cultivars; ‘Nikko’ and ‘Fukumimi’: mildly pungent pepper cultivars; ‘Fushimi’ and ‘Manganji’: nonpungent pepper cultivars. **(B)** *Pun1* gene expression in different pepper cultivars. *Pun1* transcript levels were determined using quantitative RT-PCR (means ± SE; *n* = 3) with the same RNA as that used for the *pAMT* analyses. **(C)** Capsaicin levels in pungent and nonpungent pepper cultivars as determined by HPLC.

To understand the roles of pAMT and Pun1 in capsaicinoid accumulation, we analyzed the gene expression levels of *pAMT* and *Pun1* in various pepper cultivars that differ in pungency. We extracted RNA from the placental tissues 25 daf and analyzed the expression levels of *pAMT* using quantitative RT-PCR (Figure 4A). The expression levels of *pAMT* were significantly higher in the pungent varieties than in the mildly pungent and nonpungent varieties, while *pAMT* transcripts were barely detectable in the nonpungent varieties, suggesting that the more pungent the variety, the higher the *pAMT* expression. We next measured the *Pun1* transcript levels using the same RNA used for the analyses of *pAMT*. As shown in Figure 4B, the *Pun1* transcript levels were relatively high in the pungent cultivars, but very low in the nonpungent cultivars. Just like for *pAMT*, the more pungent the cultivar, the higher the *Pun1* expression. High-performance liquid chromatography (HPLC) analyses showed that the pungent cultivars produced higher levels of capsaicin (Figure 4C). Thus, the accumulation levels of capsaicinoids seem to correlate with the expression levels of *Pun1* and *pAMT*.

**Figure 5:**
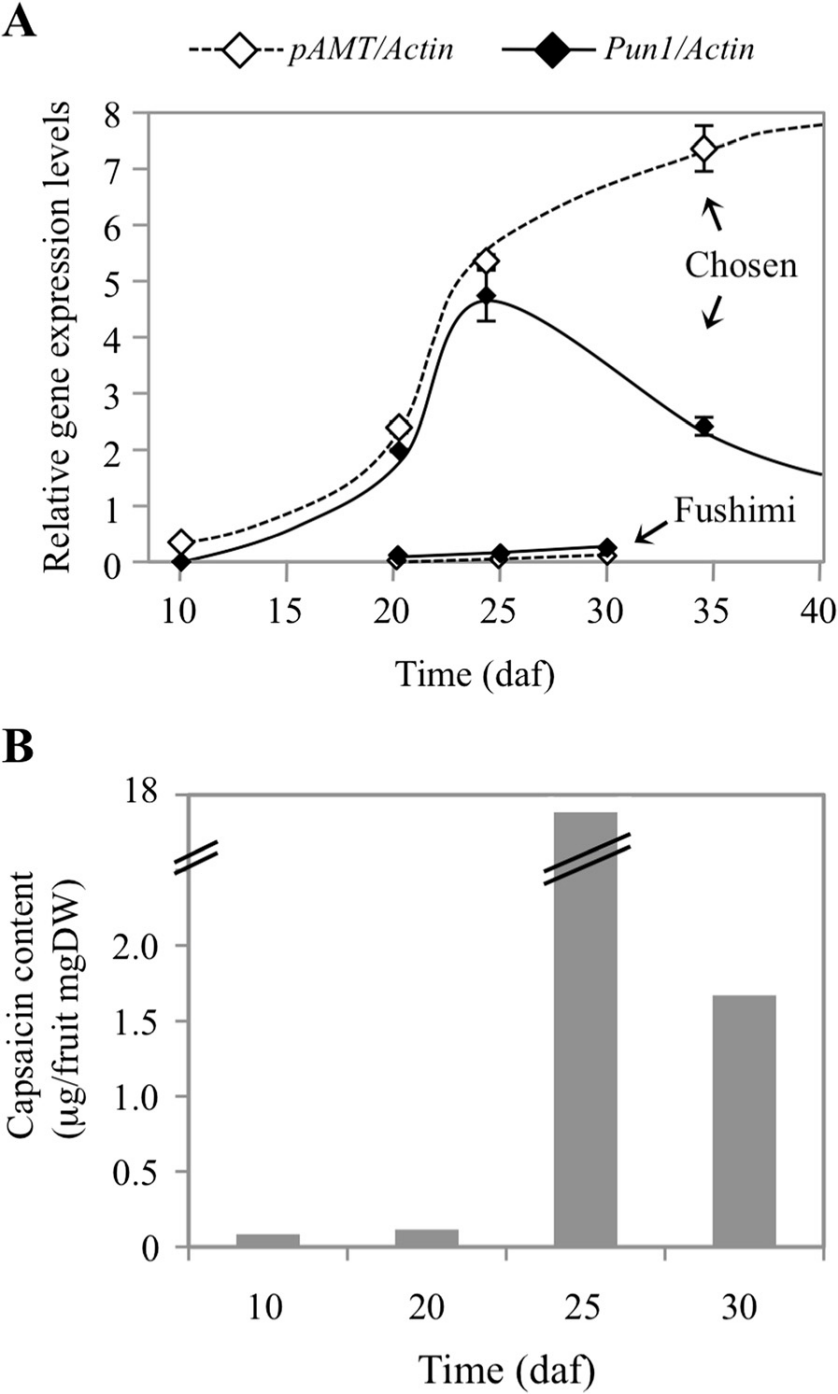
**Gene expression levels of** ***pAMT*** **and** ***Pun1*** **and capsaicin levels during fruit development. (A)** Quantitative RT-PCR to determine RNA levels of *pAMT* and *Pun1* in placental tissues (means ± SE; *n* = 3). Samples of ‘Chosen’ (pungent) were harvested at 10, 20, 25 and 35 days after flowering, and samples of ‘Fushimi’ (nonpungent) were harvested at 20, 25 and 30 days after flowering. **(B)** Capsaicin levels in the fruits of ‘Chosen’ (pungent) during fruit development as analyzed by HPLC.

When we examined the expression levels of *pAMT* and *Pun1* over time using quantitative RT-PCR (Figure 5A), as expected, the expression levels of *pAMT* and *Pun1* were very high in the pungent cultivar ‘Chosen’ but barely present in the nonpungent cultivar ‘Fushimi’. It is noteworthy that *Pun1* mRNA in ‘Chosen’ decreased after 25 daf when capsaicin reached its maximum level in the placenta (Figure 5B), whereas *pAMT* mRNA continued to increase even at 35 daf.

### Comparison of vanillylamine contents between pungent and nonpungent varieties

**Figure 6:**
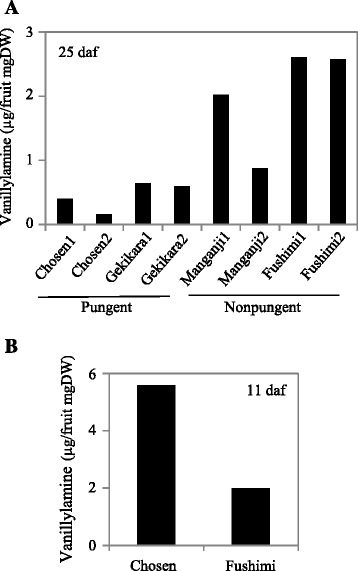
**Vanillylamine levels in pungent and nonpungent pepper cultivars. (A)** Vanillylamine levels at 25 days after flowering. **(B)** Vanillylamine levels at 11 days after flowering. Freeze-dried pepper fruits were ground, and vanillylamine was extracted with 0.1% acetic acid methanol and then analyzed by HPLC at 280 nm. The *x*-axis in **(A)** shows individual fruits. Three fruits were used for **(B)**.

To confirm that *Pun1*, rather than *pAMT*, is the primary determinant of capsaicinoid accumulation during capsaicin synthesis, we compared vanillylamine levels between pungent and nonpungent cultivars. The HPLC analyses showed significantly greater levels of vanillylamine in the placental tissues of nonpungent cultivars at 25 daf (Figure 6A). This result was unexpected because we had initially thought that the vanillylamine contents would be relatively low owing to the low *pAMT* expression in nonpungent cultivars. When we analyzed vanillylamine levels in immature placental tissues at 11 daf, vanillylamine was relatively abundant in the pungent cultivar compared with the nonpungent cultivar (Figure 6B). Thus, we assumed that most of the vanillylamine was efficiently converted to capsaicinoids by Pun1, which was present at high levels soon after vanillylamine synthesis in pungent cultivars at 25 daf. However, the very low levels of Pun1 present in nonpungent cultivars were probably not sufficient to produce capsaicin despite the high accumulation of vanillylamine. However, low levels of pAMT were still capable of converting vanillin to vanillylamine in nonpungent cultivars. Thus, we hypothesize that Pun1 is a more critical regulator of capsaicin synthesis than pAMT.

## Discussion

Previous knockdown experiments of the *pAMT* and *Pun1* genes by VIGS demonstrated that the down-regulation of these genes resulted in reduced capsaicinoid contents in fruits [13,17], indicating that both genes are responsible for the last steps in capsaicinoid synthesis. In this study, we obtained a similar result with the VIGS targeting of the *Pun1* gene using the CMV vector. The whole-genome sequencing of pepper revealed that there were three copies of the *AT3* (*Pun1*) gene [23]. According to their genome structures and fruit-specific gene expression levels, AT3-D1 and AT3-D2 may be functional in capsaicinoid biosynthesis. Because they are very similar at the 95 nt long target sequence for VIGS, we assumed that both genes were simultaneously down-regulated by VIGS. We then compared the expression levels of the *pAMT* and *Pun1* genes and the capsaicin accumulation levels between pungent and nonpungent cultivars. The quantitative RT-PCR analyses indicated that the capsaicinoid levels correlated well with the expression levels of both *pAMT* and *Pun1*, with expression of the two genes in the pungent cultivars being 50- to 100-fold higher than in the nonpungent cultivars. These results strongly suggest that capsaicinoid levels (pepper pungency) are primarily determined by the expression levels of both *pAMT* and *Pun1*, which are exclusive to the pungent cultivars. Presently, we cannot completely eliminate the possibility that the enzymatic activities of pAMT and/or Pun1 differ between pungent and nonpungent cultivars. However, it is unlikely that the enzymatic activities of pAMT and/or Pun1 are the determinants of pepper pungency over their transcriptional levels because their mRNA levels in the nonpungent cultivars ranged from extremely low to undetectable in our experiments. Thus, the enzymes per se do not accumulate in the placenta, regardless of their activity levels. The accumulation of the AT3 (Pun1) protein is reported to be closely correlated with the level of *AT3* (*Pun1*) transcripts in the pungent cultivar ‘Thai Hot’ [13]. Additionally, *Pun1* is highly expressed in the pungent cultivar *C. chinense* ‘Habanero’ at the transcript and protein levels, but was hardly detectable in the nonpungent cultivars *C. annuum* ‘Maor’ and *C. chinense* NMCA30036 [24]. These reports indicate that capsaicinoid biosynthesis may be regulated primarily at the transcription level. Although the *pAMT* expression level in the nonpungent cultivar *C. annuum* ‘CH-19 Sweet’ was reported to be comparable with that in the pungent cultivars, this would be an exceptional case because ‘CH-19 Sweet’ accumulates capsinoids instead of capsaicinoids [18].

The transamination of vanillin to vanillylamine and the acylation of vanillylamine with a branched-chain fatty acid are critical steps in the biosynthesis of capsaicinoids [10,11]. The most plausible candidate genes for the acylation, *Pun1*, and the putative aminotransferase gene, *pAMT*, have been highly correlated with capsaicinoid accumulation in several genetic studies of mutant alleles that corresponded to either *pAMT* or *Pun1* [13,18,19]. However, because the enzymatic activities of pAMT and/or Pun1 may differ between the pungent and nonpungent cultivars, we should be able to measure the enzymatic activities of the enzymes. Thus, biochemical verifications will still be necessary to determine whether these putative genes are the real players in the pathway. Recently, based on bioinformatics analyses, single nucleotide polymorphisms in the *Pun1* locus were found to distinguish pungent from nonpungent cultivars [25,26]. This suggests that *Pun1* is indeed the locus responsible for the qualitative effect on pungency in pepper cultivars and that the enzymatic activity of Pun1 may be categorized into two levels, which determine whether a cultivar will be pungent or not. Since Kim *et al*. [14] reported that cDNA clone SB2-66 is identical to the *Pun1* gene, Pun1 has been suspected to be the actual CS, but there has been no cell-free system to synthesize capsaicin. Purified proteins with CS activity have suffered from contamination with endogenous capsaicinoids; therefore, researchers must use a radioactive substrate, which is not commercially available. Here, we overcame these technical difficulties by using protoplasts isolated from placental tissues of a pungent cultivar. This protoplast-based assay is so simple and easy that comparative experiments assessing Pun1 activities among pepper cultivars are now possible. In addition, this system can be used for other purposes, such as measuring other enzymatic activities and screening for capsaicin synthesis inhibitors.

Even though the transcript levels of both *pAMT* and *Pun1* were very low in the nonpungent cultivars compared with the pungent cultivars, the vanillylamine levels were five times higher in the nonpungent cultivars than in the pungent cultivars at 25 daf (Figure 6). Although these results seem to contradict each other, one possible explanation is that the vanillylamine synthesized in the pungent cultivars was quickly converted to capsaicin by the high Pun1 enzyme activity (Additional file 4: Figure S4). According to this hypothesis, which is based on an expression time course of *pAMT* and *Pun1* relative to the vanillylamine accumulation levels, vanillylamine must be efficiently synthesized in nonpungent cultivars even when there is a very low *pAMT* expression level at 25 daf. From this point of view, the nonpungent feature is substantially determined by the loss of function of Pun1 (or very low *Pun1* expression levels), and thus the nonpungent phenotype, with relatively high vanillylamine levels, was determined essentially by low expression levels of *Pun1* but not of *pAMT*. Therefore, Pun1 would be the primary determinant of vanillylamine, as well as capsaicin, accumulation. A nonpungent cultivar *C. annuum* ‘CH-19 Sweet’ [18] was shown to accumulate less vanillylamine than pungent cultivars, but this case is exceptional because ‘CH-19 Sweet’ must have enough Pun1 activity to accumulate a high capsinoids (capsiate) content, unlike the nonpungent cultivars used in this study that showed very low *Pun1* expression levels.

## Conclusions

To verify that the *Pun1* gene is actually involved in the final step of capsaicin biosynthesis, we raised antibodies against the *E. coli*-synthesized Pun1 protein and used them as antagonists of endogenous Pun1 activity. After confirmation of the antibodies’ specificity, we developed an *in vitro* cell-free assay for *de novo* capsaicin synthesis using protoplasts from placental tissues. The addition of anti-Pun1 antibodies significantly reduced *de novo* capsaicin synthesis in the protoplast assay, demonstrating that the *Pun1*-encoded protein played an essential role in capsaicin synthesis. Analyses of the accumulation of vanillylamine, a precursor of capsaicin, revealed that nonpungent cultivars could accumulate much higher vanillylamine levels than pungent cultivars. This observation indicates that *Pun1* is the primary determinant of vanillylamine and capsaicin accumulation and is more important than *pAMT* in maintaining capsaicin-free fruit in most nonpungent cultivars.

## Methods

### Plant materials

*Capsicum annuum* (L.) cultivars ‘Chosen’, ‘Gekikara’ and ‘Kaien’ were used as the pungent cultivars, ‘Nikko’ and ‘Fukumimi’ as the mildly pungent, and ‘Fushimi’ and ‘Manganji’ as the nonpungent cultivars. *C. annuum* var. *grossum* (bell pepper) was also used as a capsaicinoid-free control pepper. Plants were cultured in an incubation room under 16 h of fluorescent light at 24°C and 50% relative humidity. Green fruits at ~25 daf were used for the experiments unless otherwise indicated.

### Cloning of the *pAMT* and *Pun1* genes

Total RNA was extracted from the placental tissues of pepper fruits using TRIZOL reagent (Ambion) according to the manufacturer’s instructions. For cloning *pAMT* and *Pun1*, total RNA extracted from ‘Chosen’ was used as a template for reverse transcription with AMV Reverse Transcriptase (NIPPON GENE). The full-length cDNAs for *pAMT* and *Pun1* were amplified by PCR with the primer set 5′-ATGGCCAATATTACTAATG-3′ and 5′-TTAATGCTTCTGAGAC-3′ and the primer set 5′-ATGGCTTTTGCATTACCATC-3′ and 5′-TTAATTAGGCAATGAACTCAAGG-3′, respectively. The pGEM-T Easy Vector System I (Promega) was used for the subcloning and subsequent sequencing of the PCR products. Sequencing was performed with the BECKMAN COULTER CEQ8800 system. We designed all primers based on published sequences of the *pAMT* (AF085149) and *Pun1* (AY819029) genes.

### Quantitative RT-PCR analysis

cDNA was synthesized from DNase-treated RNA and amplified using KOD SYBR qPCR Mix (Toyobo) according to the manufacturer’s instructions. mRNA levels were quantified by quantitative RT-PCR using the StepOne Real-Time PCR System (Applied Biosystems). The *Actin* gene was used as an internal control. Primer sets for each gene amplification were as follows: *Actin*, 5′-GGTTAAGGCTGGATTTGCTG-3′ and 5′-ATGCATCCTTTTGACCCATC-3′; *pAMT*, 5′-GTAAGTTCCACTGGTGATCATG-3′ and 5′-TTACTGCTTCTGAGAC-3′; and *Pun1*, 5′-AGGCATCATCAATGCTAC-3′ and 5′-ATGTTAGTTGCTTCTATGGAG-3′.

### Virus-induced gene silencing (VIGS) against *Pun1*

The *Cucumber mosaic virus* (CMV) vector (CMV-A1 vector) had been used previously for VIGS experiments in pepper plants [27]. Here, we modified the CMV-A1 vector by changing an R residue at position 46 into a C residue in the 2b protein (2b), which weakened the RNA silencing suppressor activity of 2b so that the viral symptoms became milder in the pepper. The modified CMV-A1 vector was designated CMV-Yd. We used a pungent pepper cultivar ‘Kaien’ for the VIGS experiments because CMV-Yd can systemically infect ‘Kaien’ and easily penetrate into placental tissues. A 95-bp fragment from the *Pun1* gene was amplified by the primer pair 5′-CGCACGCGTGAAGGAAGTTGAGGTGGCATA-3′ and 5′-CGAGGCCTGAGCAGTTTCCCTTCTCTCATTG-3′ and inserted between the *Sma*I and *Mlu*I sites in the CMV-Yd vector to create CMV-Yd:CS95.

### Extraction of vanillylamine and capsaicin

Fruits were harvested, frozen and dried completely in a freeze-dryer (FDU-1200, EYELA) for 2 d. Dried fruits were then ground in a blender (Microsmash MS100, TOMY) at room temperature. For the vanillylamine extraction, 1 ml of 0.1% acetic acid methanol was added to 100 mg of dry fruit powder. For the capsaicin extraction, 0.1% acetic acid acetonitrile was added instead. After the samples were mixed, they were allowed to settle for 1 h at room temperature. The supernatant was passed through a 0.2 μm-pore membrane filter before being used in HPLC (JASCO LC-2000 system).

### HPLC analysis

The samples were separated in a SHIM-PACK VP-ODS column (150 mm × 4.6 mm; Shimazu). For vanillylamine, the eluent was a mixture of methanol/10 mM phosphate buffer pH 2.6 (3:2 v/v), and the flow rate was 0.8 ml/min. For capsaicin, the eluent was a mixture of acetonitrile/1% acetic acid (2:1 v/v), and the flow rate was 1 ml/min. All eluates were monitored at 280 nm using a UV detector. External standards were prepared by dissolving commercial vanillylamine (Sigma-Aldrich) and capsaicin (Sigma-Aldrich) in methanol and acetonitrile, respectively.

### *In vitro* cell-free assay for capsaicin synthesis

Protoplasts were isolated from fruits of the pungent cultivar ‘Chosen’ as previously described [28], except that cells were not shaken during the enzyme digestion and the reaction was stopped when the mass of cells (roughly 20–30 cells/mass) were released from the tissues. A 500-μl sample of the protoplasts was transferred to a 1.5-ml tube and centrifuged (3 min, 100 × *g*, 4°C) to remove the supernatant. Protoplasts were then resuspended in 200 μl solution containing 1 mM aqueous vanillylamine (Sigma-Aldrich). Protoplasts were ruptured to release cellular enzymes, including CS, by vortexing the mixture 4 times for 5 s each. After a 2-h incubation at room temperature, followed by centrifugation (2 min, 6,000 × *g*, 4°C) of the reaction mixture, the supernatant was collected and extracted with the ethyl acetate. The extract was dried overnight at room temperature in the dark, resuspended in 100 μl of 0.1% acetic acid acetonitrile and then passed through a 0.2-μm-pore membrane filter before HPLC. To inhibit CS activity, anti-Pun1 antibodies (described next) were added to the reaction mixture at a final dilution of 1:2000 before the *in vitro* capsaicin synthesis started.

### Expression of Pun1 in *E. coli* and production of anti-Pun1 antibodies

The open-reading frame of the *Pun1* gene was amplified with primer set 5′-CGGAATTCATGGCTTTTGCATTACCATC-3′ containing an *Eco*RI site and 5′-CGGGATCCTAATTAGGCAATGAACTCAAGG-3′ containing a *Bam*HI site, and cloned between the *Eco*RI and *Bam*HI sites of pMAL-c2x (New England Biolabs). The N-terminal maltose binding protein (MBP)-fused Pun1 recombinant protein was then expressed in *E. coli* (BL21) and affinity-purified using the pMAL Protein Fusion and Purification System (New England BioLabs). Anti-Pun1 antibodies, prepared by Frontier Institute Co. (Sapporo, Japan), were raised by immunizing a rabbit with the purified recombinant protein.

### Western blots

Total proteins from pepper placentas, extracted as described by Masuta *et al.* [29], were separated on 10% sodium dodecyl sulfate polyacrylamide gels (SDS-PAGE) and blotted on an Immobilon-P membrane (Millipore). The blots were then treated with primary antibodies raised against the *E. coli*-synthesized Pun1 protein, and subsequently with goat anti-rabbit IgG-alkaline phosphatase-conjugate (Takara Bio) as a secondary antibody. Signals were visualized using CDP-Star (Roche) as a substrate.

## Additional files

Additional file 1: Figure S1. Western blot analysis of the *in vitro* synthesized pepper hydroxycinnamoyl transferase (HCT) protein using the anti-Pun1 antibodies. The cDNA clone of HCT was PCR-amplified and inserted in the pEU plasmid vector (CellFree Sciences, Japan). After *in vitro* transcription from the recombinant plasmid, HCT-fused to a C-terminal FLAG peptide (HCT-FLAG) was *in vitro* synthesized in the wheat germ cocktail (CellFree Sciences, Japan) according to the manufacturer’s instructions. The HCT protein with a FLAG tag was then subjected to detection either by the anti-Pun1 antibodies (left) or by an anti-FLAG antibody (right). Note that anti-Pun1 antibodies did not react with the HCT protein. The arrow indicates the HCT protein. Additional file 2: Figure S2. Accumulation levels of capsaicinoids in placental tissues infected with CMV-Yd:CS95. The graph shows the data as fold change compared with the healthy control (means ± SD from three separate experiments using 3 to 5 pepper fruits for each treatment). The reduction in capsaicin accumulation in CMV-Yd:CS95 was statistically significant compared with the empty vector control (CMV-Yd) using Student’s t-test. Asterisks indicate significance at 0.01. Additional file 3: Figure S3. HPLC chromatograms of *de novo* capsaicin synthesis products from the *in vitro* cell-free assay using protoplasts. The main peak (red) in each chart represents capsaicin. This experiment was repeated twice and produced the same results. Additional file 4: Figure S4. Hypothesis to explain the accumulation of vanillylamine in nonpungent pepper cultivars. In pungent peppers, vanillylamine is quickly converted to capsaicin by highly active CS, while in nonpungent cultivars, vanillylamine is synthesized even with a very low pAMT activity level. Although vanillylamine is more abundant in nonpungent cultivars than in the pungent cultivars at 25 daf (dotted line), it will not be converted to capsaicin owing to the very low level of CS activity. This result is supported by the gene expression time-course of *pAMT* and *Pun1* (Figure 5).

## Abbreviations

CS: Capsaicin synthase
daf: Days after flowering
pAMT: Putative aminotransferase
Pun1: Pungent gene 1
HCT: Hydroxycinnamoyl transferase
VIGS: Virus-induced gene silencing
