# Supplementary material for: Evidence of capsaicin synthase activity of the Pun1-encoded protein and its role as a determinant of capsaicinoid accumulation in pepper
Source: BMC Plant Biol. 2015 Mar 28;15:93. doi: 10.1186/s12870-015-0476-7 (PMC4386094; doi:10.1186/s12870-015-0476-7)
Supplement: Additional file 1: Figure S1. — Western blot analysis of the in vitro synthesized pepper hydroxycinnamoyl transferase (HCT) protein using the anti-Pun1 antibodies. The cDNA clone of HCT was PCR-amplified and inserted in the pEU plasmid vector (CellFree Sciences, Japan). After in vitro transcription from the recombinant plasmid, HCT-fused to a C-terminal FLAG peptide (HCT-FLAG) was in vitro synthesized in the wheat germ cocktail (CellFree Sciences, Japan) according to the manufacturer’s instructions. The HCT protein with a FLAG tag was then subjected to detection either by the anti-Pun1 antibodies (left) or by an anti-FLAG antibody (right). Note that anti-Pun1 antibodies did not react with the HCT protein. The arrow indicates the HCT protein. [file 12870_2015_476_MOESM1_ESM.pdf]

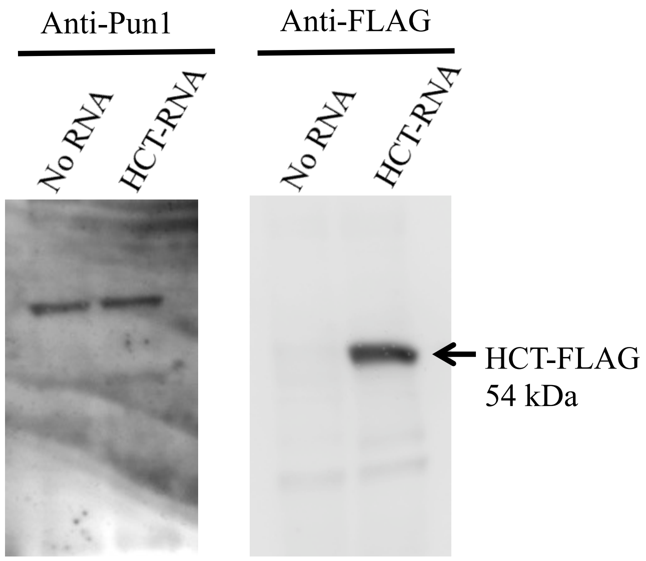

Figure S1. Western blot analysis of the in vitro synthesized pepper hydroxycinnamoyl transferase (HCT) protein using the anti-Pun1 antibodies. The cDNA clone of HCT was PCR-amplified and inserted in the pEU plasmid vector (CellFree Sciences, Japan). After in vitro transcription from the recombinant plasmid, HCT-fused to a C-terminal FLAG peptide (HCT-FLAG) was in vitro synthesized in the wheat germ cocktail (CellFree Sciences, Japan) according to the manufacturer's instructions. The HCT protein with a FLAG tag was then subjected to detection either by the anti-Pun1 antibodies (left) or by an anti-FLAG antibody (right). Note that anti-Pun1 antibodies did not react with the HCT protein. The arrow indicates the HCT protein.
