# Supplementary material for: Evidence of capsaicin synthase activity of the Pun1-encoded protein and its role as a determinant of capsaicinoid accumulation in pepper
Source: BMC Plant Biol. 2015 Mar 28;15:93. doi: 10.1186/s12870-015-0476-7 (PMC4386094; doi:10.1186/s12870-015-0476-7)
Supplement: Additional file 2: Figure S2. — Accumulation levels of capsaicinoids in placental tissues infected with CMV-Yd:CS95. The graph shows the data as fold change compared with the healthy control (means ± SD from three separate experiments using 3 to 5 pepper fruits for each treatment). The reduction in capsaicin accumulation in CMV-Yd:CS95 was statistically significant compared with the empty vector control (CMV-Yd) using Student’s t-test. Asterisks indicate significance at 0.01. [file 12870_2015_476_MOESM2_ESM.pdf]

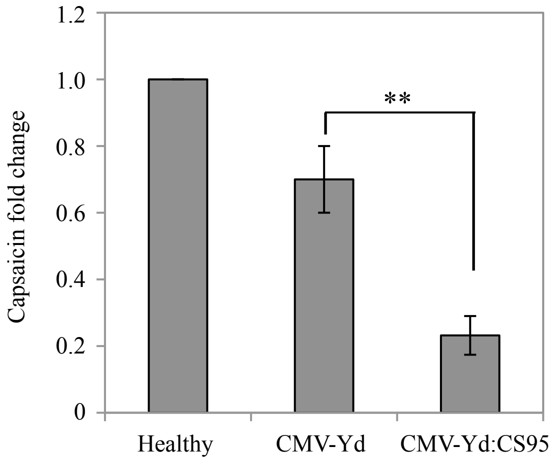

Figure S2. Accumulation levels of capsaicinoids in placental tissues infected with CMV-Yd:CS95. The graph shows the data as fold change compared with the healthy control (means ± SD from three separate experiments using 3 to 5 pepper fruits for each treatment). The reduction in capsaicin accumulation in CMV-Yd:CS95 was statistically significant compared with the empty vector control (CMV-Yd) using Student's t-test. Asterisks indicate significance at 0.01.
