# Supplementary material for: Evidence of capsaicin synthase activity of the Pun1-encoded protein and its role as a determinant of capsaicinoid accumulation in pepper
Source: BMC Plant Biol. 2015 Mar 28;15:93. doi: 10.1186/s12870-015-0476-7 (PMC4386094; doi:10.1186/s12870-015-0476-7)
Supplement: Additional file 3: Figure S3. — HPLC chromatograms of de novo capsaicin synthesis products from the in vitro cell-free assay using protoplasts. The main peak (red) in each chart represents capsaicin. This experiment was repeated twice and produced the same results. [file 12870_2015_476_MOESM3_ESM.pdf]

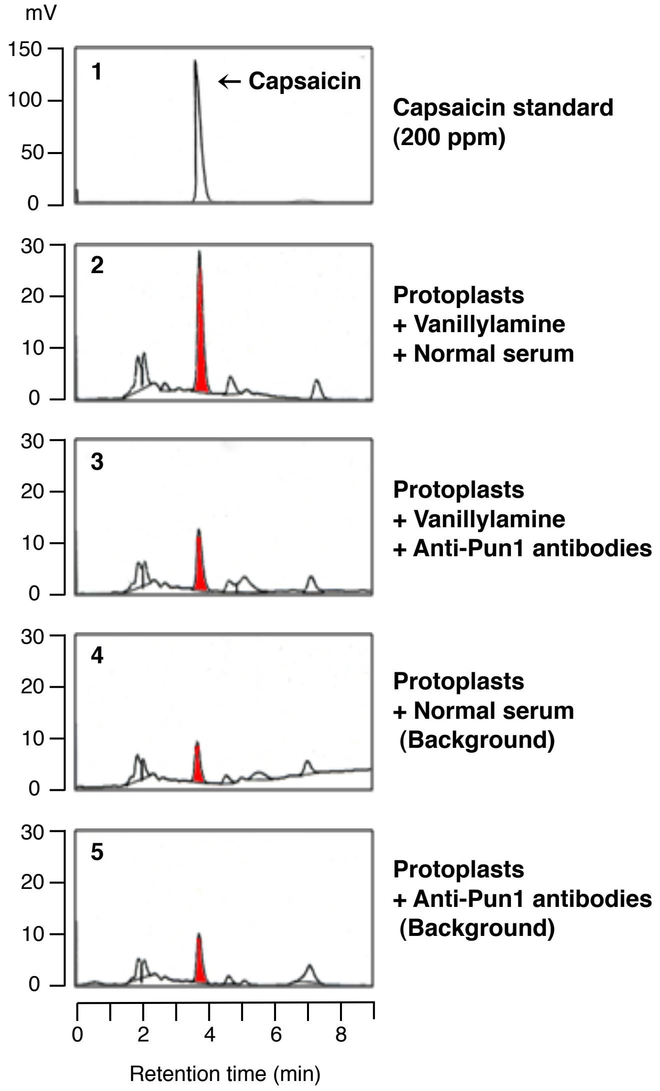

Figure S3. HPLC chromatograms of de novo capsaicin synthesis products from the in vitro cell-free assay using protoplasts. The main peak (red) in each chart represents capsaicin. This experiment was repeated twice and produced the same results.
