# Supplementary material for: Evidence of capsaicin synthase activity of the Pun1-encoded protein and its role as a determinant of capsaicinoid accumulation in pepper
Source: BMC Plant Biol. 2015 Mar 28;15:93. doi: 10.1186/s12870-015-0476-7 (PMC4386094; doi:10.1186/s12870-015-0476-7)
Supplement: Additional file 4: Figure S4. — Hypothesis to explain the accumulation of vanillylamine in nonpungent pepper cultivars. In pungent peppers, vanillylamine is quickly converted to capsaicin by highly active CS, while in nonpungent cultivars, vanillylamine is synthesized even with a very low pAMT activity level. Although vanillylamine is more abundant in nonpungent cultivars than in the pungent cultivars at 25 daf (dotted line), it will not be converted to capsaicin owing to the very low level of CS activity. This result is supported by the gene expression time-course of pAMT and Pun1 (Figure 5). [file 12870_2015_476_MOESM4_ESM.pdf]

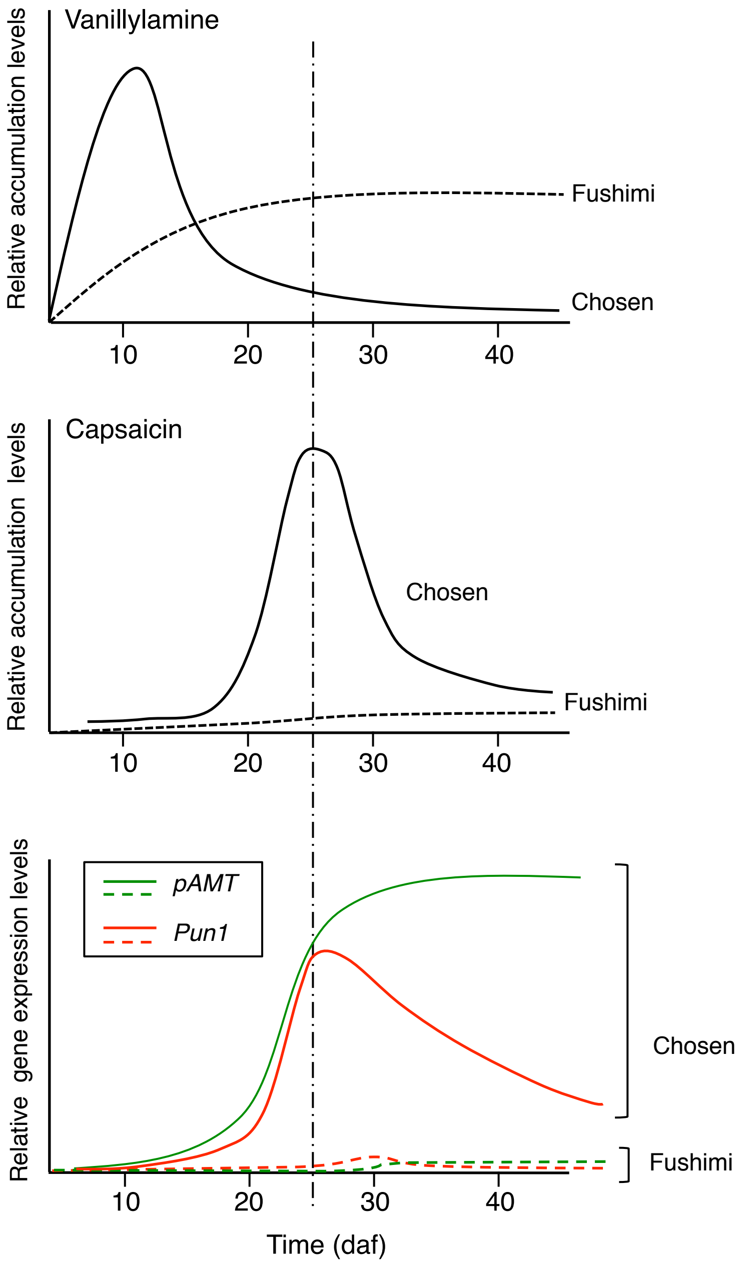

Figure S4. Hypothesis to explain the accumulation of vanillylamine in nonpungent pepper cultivars. In pungent peppers, vanillylamine is quickly converted to capsaicin by highly active CS, while in nonpungent cultivars, vanillylamine is synthesized even with a very low pAMT activity level. Although vanillylamine is more abundant in nonpungent cultivars than in the pungent cultivars at 25 daf (dotted line), it will not be converted to capsaicin owing to the very low level of CS activity. This result is supported by the gene expression time-course of pAMT and Pun1 (Figure 5).
